# Supplementary material for: The dominantly expressed class II molecule from a resistant MHC haplotype presents only a few Marek’s disease virus peptides by using an unprecedented binding motif
Source: PLoS Biol. 2021 Apr 26;19(4):e3001057. doi: 10.1371/journal.pbio.3001057 (PMC8101999; doi:10.1371/journal.pbio.3001057)
Supplement: S1 Fig — Comparison of peptides (A) from all CVI988 samples pooled and from all RB-1B samples pooled, (B) from CVI988 sample and RB-1B sample from 2019 experiment, (C) from CVI988 sample and RB-1B sample from 2020 experiment, (D) from CVI988 samples in 2019 and 2020 experiments, and (E) from RB-1B samples in 2018, 2019, and 2020 experiments. Total peptide numbers are below each sample name; percentages indicate peptides unique to a particular sample (that is, not shared) in a particular comparison and are rounded. The underlying data for this figure can be found in S1–S6 Data. (PDF) [file pbio.3001057.s001.pdf]

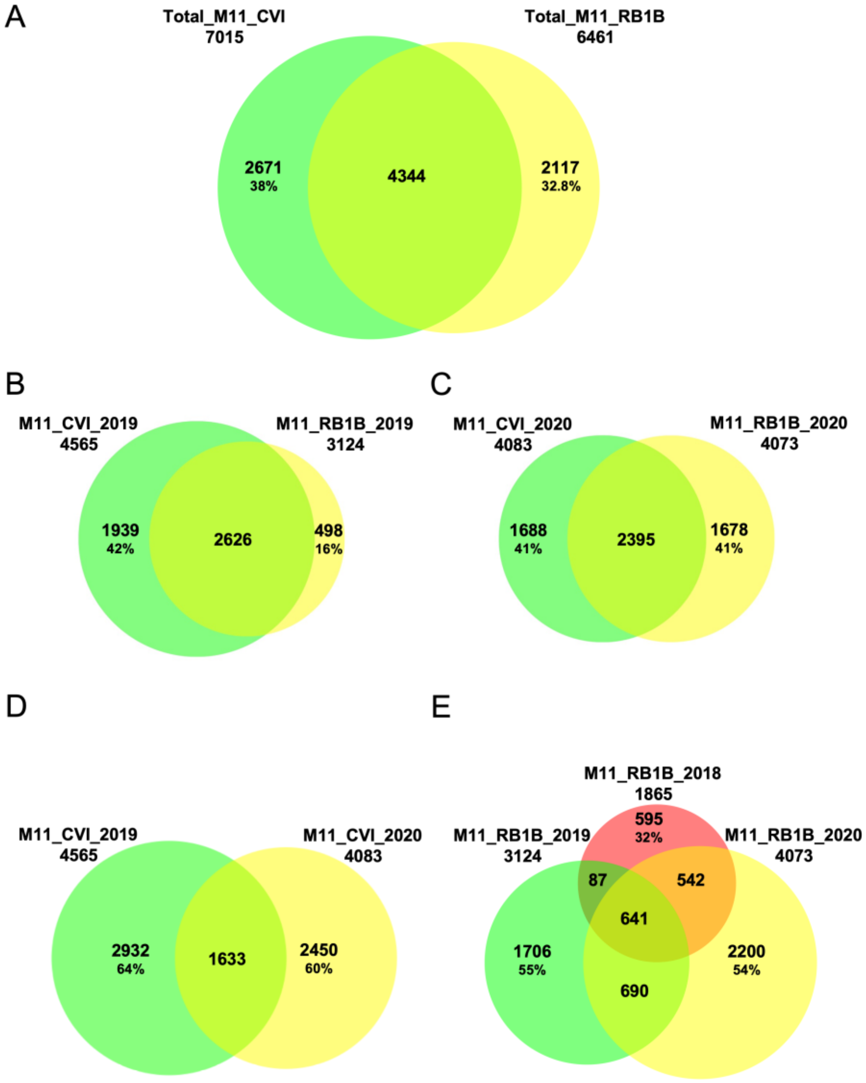


**S1 Fig.** There is a large overlap in peptides found in different samples, presented as Venn diagrams. Comparison of peptides A. from all CVI988 samples pooled and from all RB-1B samples pooled, B. from CVI988 sample and RB-1B sample from 2019 experiment, C. from CVI988 sample and RB-1B sample from 2020 experiment, D. from CVI988 samples in 2019 and 2020 experiments, E. from RB-1B samples in 2018, 2019 and 2020 experiments. Total peptide numbers are below each sample name; percentages indicate peptides unique to a particular sample (that is, not shared) in a particular comparison, and are rounded. The underlying data for this figure can be found in S1-S6 Data.
