# Supplementary material for: The dominantly expressed class II molecule from a resistant MHC haplotype presents only a few Marek’s disease virus peptides by using an unprecedented binding motif
Source: PLoS Biol. 2021 Apr 26;19(4):e3001057. doi: 10.1371/journal.pbio.3001057 (PMC8101999; doi:10.1371/journal.pbio.3001057)
Supplement: S2 Fig — Top panel, absolute numbers of peptides for each length; bottom panel, percentage of total peptides for each length. The underlying data for this figure can be found in S1–S6 Data. (PDF) [file pbio.3001057.s002.pdf]

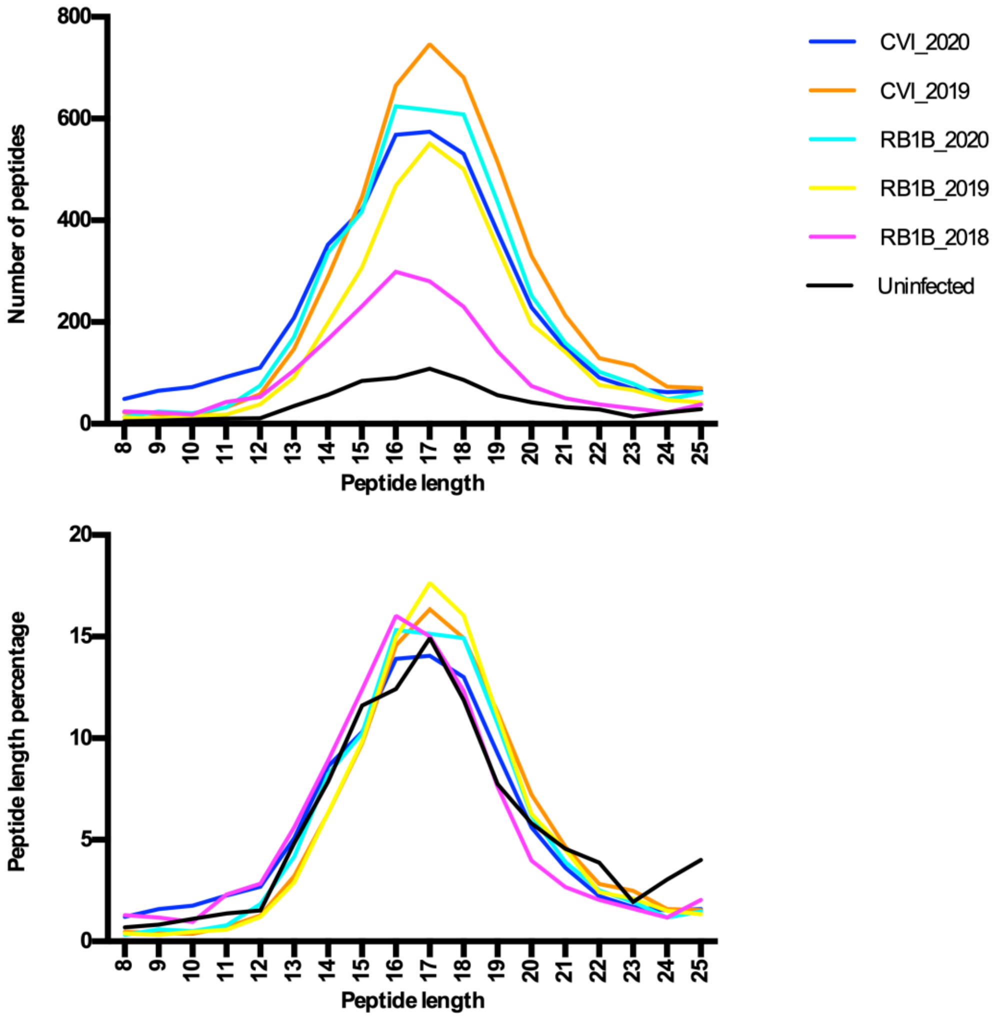

S2 Fig. Length distribution of peptides found in the six samples. Top panel, absolute numbers of peptides for each length; bottom panel, percentage of total peptides for each length. The underlying data for this figure can be found in S1-S6 Data.
