# Supplementary material for: The dominantly expressed class II molecule from a resistant MHC haplotype presents only a few Marek’s disease virus peptides by using an unprecedented binding motif
Source: PLoS Biol. 2021 Apr 26;19(4):e3001057. doi: 10.1371/journal.pbio.3001057 (PMC8101999; doi:10.1371/journal.pbio.3001057)
Supplement: S3 Fig — Shown are portions of the genomic sequences of HLA-DRB1 (AM910430 [80]) and BLB2 (called BLBII in M29763 [43]), similar to sequences from other class II molecules ([34] and other papers cited therein), aligned with the N-terminal sequences of DR1 β chain [81], similar to other human class II β chains (reviewed in [82]). Nucleotide sequences in lower case along with numbering from GenBank flat files (AM910430 for HLA-DRB1, M29763 for BLB2) with introns in lower case italic, invariant residues at start and end of introns in bold and underlined. Protein sequences in capital letters either from translation of genomic sequence or N-terminal amino acid sequencing, with signal sequence cleavage site double underlined (60% probability from SignalP-5.0, www.cbs.dtu.dk/services/SignalP/) and possible downstream signal sequence cleavage site single underlined. (PDF) [file pbio.3001057.s003.pdf]

```
N-terminal                G  D  T  R  P
AM910430      L  A  L  A  G  D  T  R  P
AM910430    cactggctttggctggggacaccagacgtaagtgcacattgtgggtgctg   50
M29763      ggccggccgccggcacgcggccctcgggtgagctcggagccgcggcgcgg  941
M29763        P  A  A  G  T  R  P  S  A
```

```
N-terminal                   R  S  L  W  Q  L  K  F  E  C  H/Y
AM910430                     R  F  L  E  E  V  R  F  E  C  H
AM910430    ttcgtgtccccacagcacgtttcttggaggaggttaagtttgagtgtcat   8050
M29763      gccctctgcccgcagcgttcttcttctgcggtgcgatatccgagtgccac   1161
M29763                       F  F  F  C  G  A  I  S  E  C  H
```

S3 Fig. The N-termini of the class II β chains of humans and chickens are in the same position. Shown are portions of the genomic sequences of HLA-DRB1 (AM910430, [80]) and BLB2 (called BLBII in M29763, [43]), similar to sequences from other class II molecules ([34] and other papers cited therein), aligned with the N-terminal sequences of DR1 β chain [81], similar to other human class II β chains (reviewed in [82]). Nucleotide sequences in lower case along with numbering from GenBank flat files (AM910430 for HLA-DRB1, M29763 for BLB2) with introns in lower case italic, invariant residues at start and end of introns in bold and underlined. Protein sequences in capital letters either from translation of genomic sequence or N-terminal amino acid sequencing, with signal sequence cleavage site double underlined (60% probability from SignalP-5.0, www.cbs.dtu.dk/services/SignalP/) and possible downstream signal sequence cleavage site single underlined.

34. Pharr GT, Dodgson JD, Hunt HH, Bacon LD. Class II MHC cDNAs in 15I5 B-congenic chickens. Immunogenetics. 1998; 47: 350-4. doi: 10.1007/s002510050369. PMID: 9510552.

43. Zoorob R, Behar G, Kroemer G, Auffray C. Organization of a functional chicken class II B gene. Immunogenetics. 1990; 31; 179-87. doi: 10.1007/BF00211553. PMID: 1969383.

80. Doxiadis GG, de Groot N, de Groot G, Doxiadis II, Bontrop RE. Reshuffling of ancient peptide binding motifs between HLA-DRB multigene family members: old wine served in new skins. Mol Immunol. 2008; 45: 2743-51. doi: 10.1016/j.molimm.2008.02.017. PMID: 18395261.

81. Walker LE, Hewick R, Hunkapiller MW, Hood LE, Dreyer WJ, Reisfeld RA. N-terminal amino acid sequences of the α and β chains of HLA-DR and HLA-DR2 antigens. Biochemistry. 1983; 22: 185-8. doi: 10.1021/bi00270a027. PMID: 6600932.

82. Kaufman JF, Auffray CA, Korman AJ, Shackelford DA, Strominger JL. The class II molecules of the human and murine major histocompatibility complex. Cell. 1984; 36: 1-13. doi: 10.1016/0092-8674(84)90068-0. PMID: 6198089.
