# Supplementary material for: The dominantly expressed class II molecule from a resistant MHC haplotype presents only a few Marek’s disease virus peptides by using an unprecedented binding motif
Source: PLoS Biol. 2021 Apr 26;19(4):e3001057. doi: 10.1371/journal.pbio.3001057 (PMC8101999; doi:10.1371/journal.pbio.3001057)
Supplement: S4 Fig — Upper panel, class II α chains; lower panel, class II β chains. Single letter amino acid code; blue, identical residues; green box, CD4 contact; yellow box, DM contact; grey box, hydrophobic transmembrane region; blue box, cytoplasmic tail. Arrow, β-strand; blue cylinder, α-helix; purple cylinder, 310-helix (secondary structure elements as determined by PyMol); red “SS,” cysteine for intrachain disulfide bond; orange “g,” glycosylation site. The underlying data for this figure can be found in PDB files 1DLH, 6KVM, and 6T3Y. (PDF) [file pbio.3001057.s004.pdf]

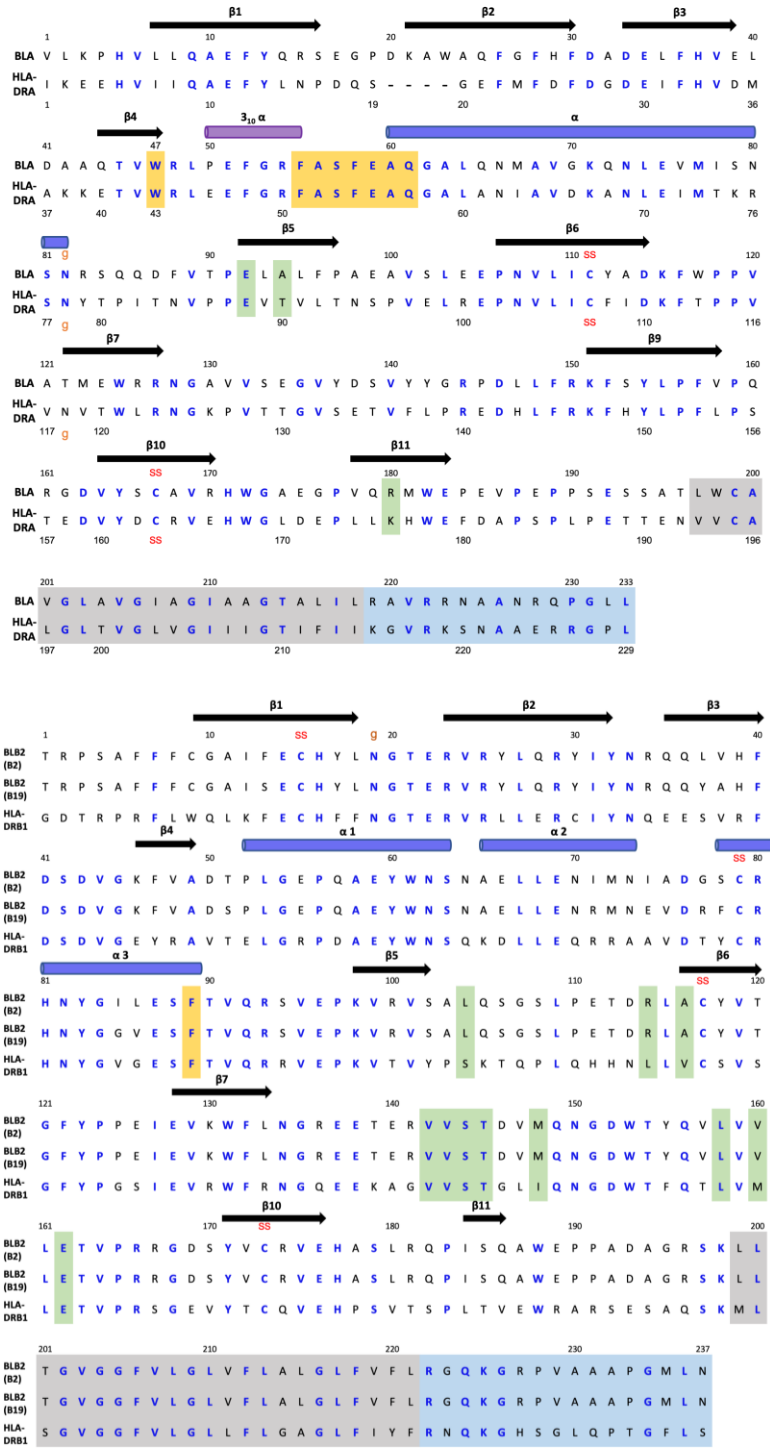


**S4 Fig.** Key residues in chicken and human class II molecules are identical or similar. Upper panel, class II α chains; lower panel, class II β chains. Single letter amino acid code; blue, identical residues; green box, CD4 contact; yellow box, DM contact; grey box, hydrophobic transmembrane region; blue box, cytoplasmic tail. Arrow, β-strand; blue cylinder, α-helix; purple cylinder, $3_{10}$-helix (secondary structure elements as determined by PyMol); red “SS”, cysteine for intrachain disulfide bond; orange “g”, glycosylation site. The underlying data for this figure can be found in PDB files 1DLH, 6KVM and 6T3Y.
