# Supplementary material for: The dominantly expressed class II molecule from a resistant MHC haplotype presents only a few Marek’s disease virus peptides by using an unprecedented binding motif
Source: PLoS Biol. 2021 Apr 26;19(4):e3001057. doi: 10.1371/journal.pbio.3001057 (PMC8101999; doi:10.1371/journal.pbio.3001057)
Supplement: S5 Fig — Comparison of BL2*02 (PDB file 6T3Y) in upper panel with DR1 (1T5W) in lower panel for (A) top view of solvent-accessible surface of class II molecules calculated by APBS electrostatics (positive charge, blue; negative charge, red) with P1 and P10 pockets for BL2*02 indicated (upper panel) and the P1 and P9 pockets along with the P10 shelf for DR1 indicated (lower panel), and with peptides in sticks, (B) side view of peptide in sticks with amino acids indicated and with pockets as surfaces with P1 and P10 pockets indicated for BL2*02, and with length between Cα of P1 and P9 or P10 shown (blue asterisks indicate that the side chains are not resolved), and (C) edge-on view of peptide Cα backbone, with arrow indicating departure of P4 in BL2*02 from the polyproline II helix. (D) Ribbon representation of class II molecule with superimposing peptide Cα backbones for BL2*02 (slate blue) and DR1 (1T5W, grey) showing top view (α1 domain above, β1 domain below) and side view (α1 domain shown, β1 domain removed for clarity), and with blue arrow indicating departure of P4 in BL2*02 from the polyproline II helix. The underlying data for this figure can be found in PDB files 1T5W and 6T3Y. (PDF) [file pbio.3001057.s005.pdf]

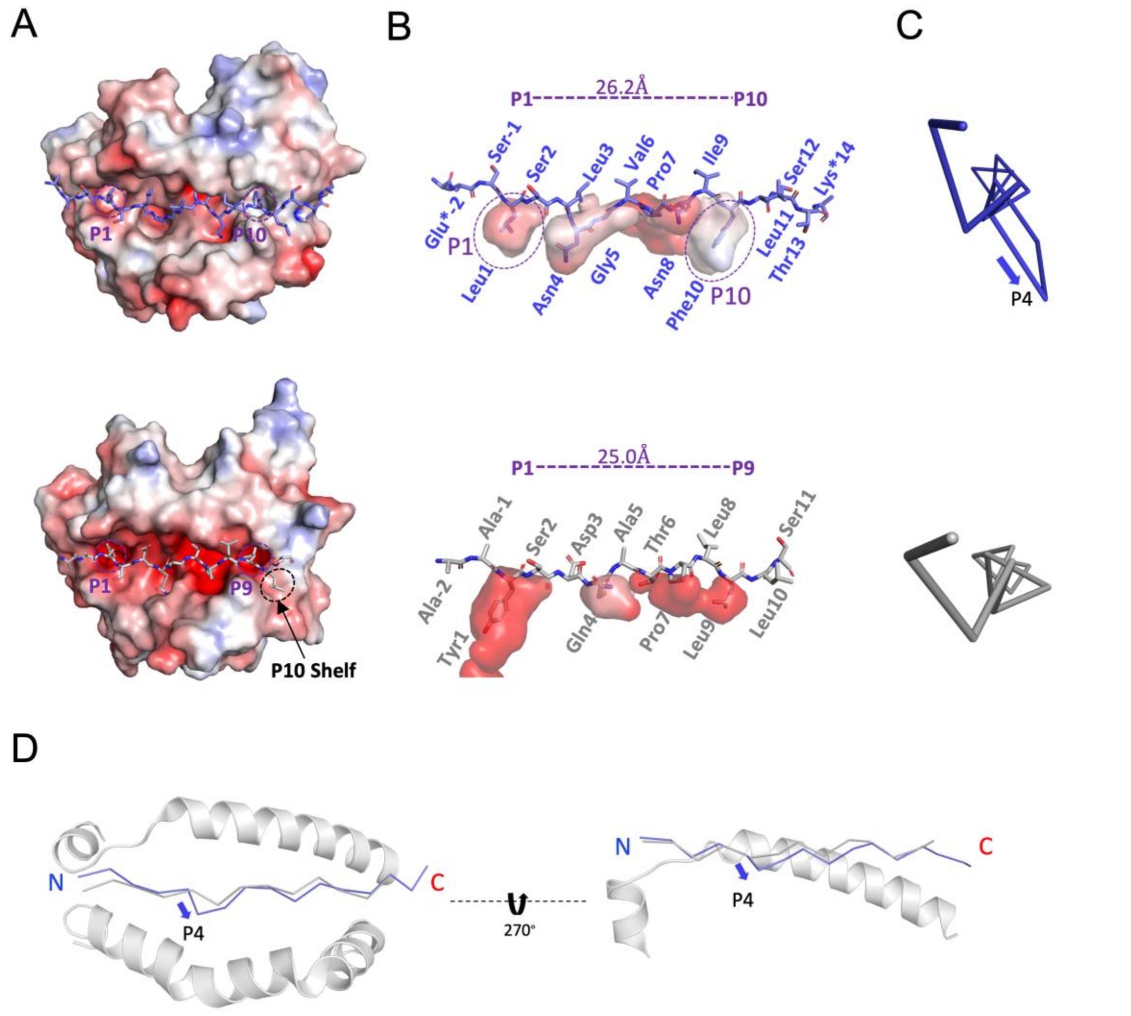

S5 Fig. The only example reported for peptide position P10 binding a class II molecule is not a deep pocket but a shelf, which is outside of the groove that binds the nonamer core. Comparison of BL2*02 (PDB file 3T6Y) in upper panel with DR1 (1T5W) (49) in lower panel for A. top view of solvent-accessible surface of class II molecules calculated by APBS electrostatics (positive charge, blue; negative charge, red) with P1 and P10 pockets for BL2*02 indicated (upper panel) and the P1 and P9 pockets along with the P10 shelf for DR1 indicated (lower panel), and with peptides in sticks, B. side view of peptide in sticks with amino acids indicated and with pockets as surfaces with P1 and P10 pockets indicated for BL2*02, and with length between Cα of P1 and P9 or P10 shown (blue asterisks indicate that the side chains are not resolved), and C. edge-on view of peptide Cα backbone, with arrow indicating departure of P4 in BL2*02 from the polyproline II helix. D. ribbon representation of class II molecule with superimposing peptide Cα backbones for BL2*02 (slate blue) and DR1 (1T5W, grey) showing top view (α1 domain above, β1 domain below) and side view (α1 domain shown, β1 domain removed for clarity), and with blue arrow indicating departure of P4 in BL2*02 from the polyproline II helix. The underlying data for this figure can be found in PDB files 1T5W and 6T3Y.

49. Zavala-Ruiz Z, Strug I, Anderson M, Gorski J, Stern L. A polymorphic pocket at the P10position contributes to peptide binding specificity in class II MHC proteins. Chem Biol. 2004;11: 1395-1402. doi: 10.1016/j.chembiol.2004.08.007. PMID: 15489166.
