# Supplementary material for: The dominantly expressed class II molecule from a resistant MHC haplotype presents only a few Marek’s disease virus peptides by using an unprecedented binding motif
Source: PLoS Biol. 2021 Apr 26;19(4):e3001057. doi: 10.1371/journal.pbio.3001057 (PMC8101999; doi:10.1371/journal.pbio.3001057)
Supplement: S6 Fig — H-bonds (dotted lines, cut-off of 4 Å) between class II molecules (ribbons with side chains of key interacting residues in brown sticks, with residue numbers in green), peptide (purple) and water molecules (blue circles), as well as hydrophobic contacts (red spokes with residue numbers in black), as determined by LigPlot+ v.2.2 (www.ebi.ac.uk/thornton-srv/software/LigPlus/), based on structures 1DLH for DR1*01, 6KVM for BL*19, and (A) 6T3Y for BL*2; (B) 6KVM for BL*19; (C) 1DLH for DR1*01; and (D) 4X5W also for DR1*01. The underlying data for this figure can be found in PDB files 1DLH, 4X5W, 6KVM, and 6T3Y. (PDF) [file pbio.3001057.s006.pdf]

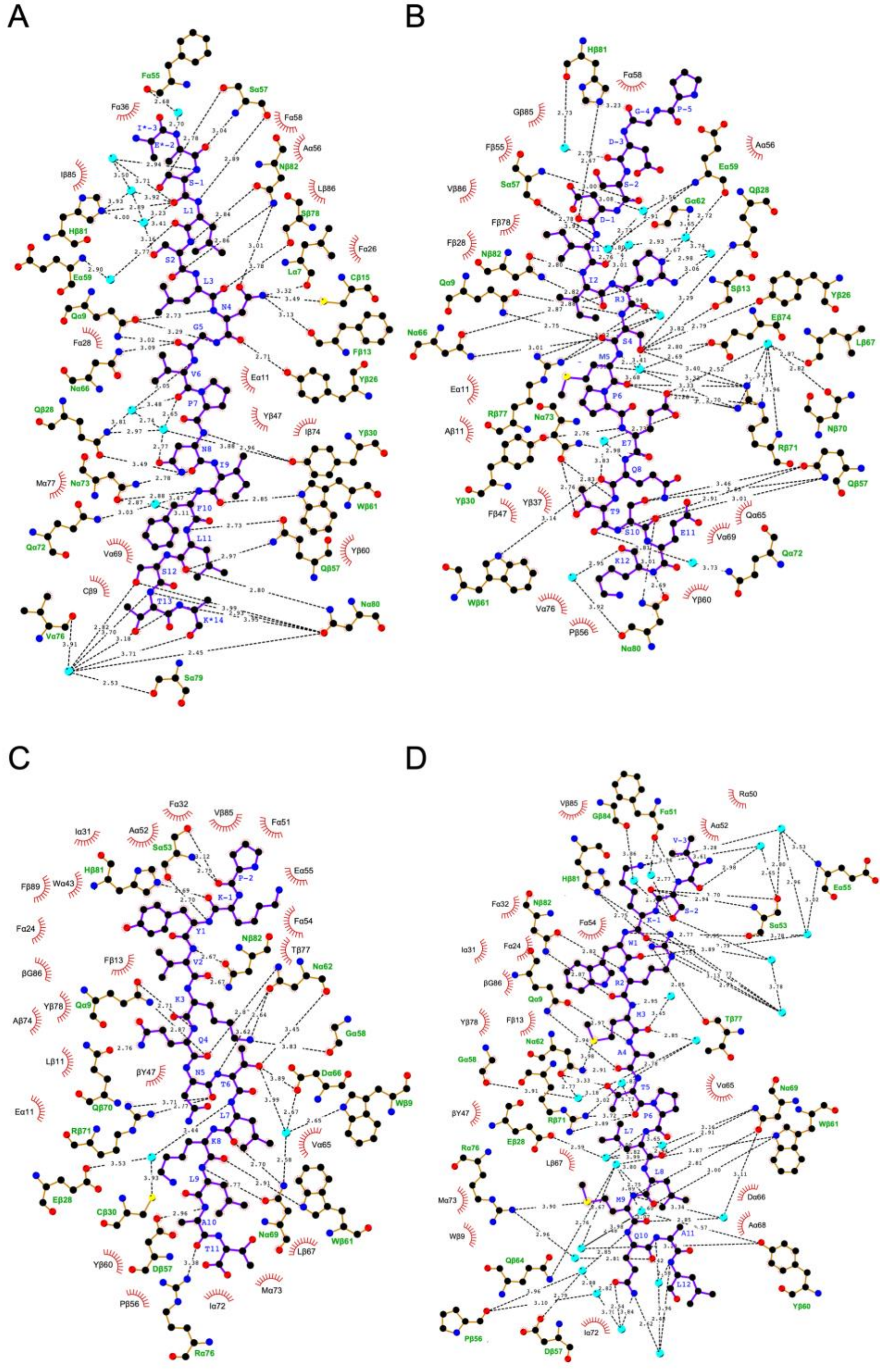

S6 Fig. H-bonds (dotted lines, cut-off of 4 Å) between class II molecules (ribbons with side chains of key interacting residues in brown sticks, with residue numbers in green), peptide (purple) and water molecules (blue circles), as well as hydrophobic contacts (red spokes with residue numbers in black), as determined by LigPlot+ v.2.2 (www.ebi.ac.uk/thornton-srv/software/LigPlus/), based on structures 1DLH for DR1*01, 6KVM for BL*19 and A. 6T3Y for BL*2; B. 6KVM for BL*19; C. 1DLH for DR1*01 and D. 4X5W also for DR1*01. The underlying data for this figure can be found in PDB files 1DLH, 6KVM, 6T3Y and 4X5W.
