# Supplementary material for: The dominantly expressed class II molecule from a resistant MHC haplotype presents only a few Marek’s disease virus peptides by using an unprecedented binding motif
Source: PLoS Biol. 2021 Apr 26;19(4):e3001057. doi: 10.1371/journal.pbio.3001057 (PMC8101999; doi:10.1371/journal.pbio.3001057)
Supplement: S7 Fig — Top panel, BL2*02 (6T3Y) with peptide in blue; middle panel, BL2*19 (6KVM) with peptide in yellow; bottom panel, HLA-DR1*01 (1DLH) with peptide in grey. Based on LigPlot analyses in S6 Fig. The underlying data for this figure can be found in PDB files 1DLH, 4X5W, 6KVM, and 6T3Y. (PDF) [file pbio.3001057.s007.pdf]

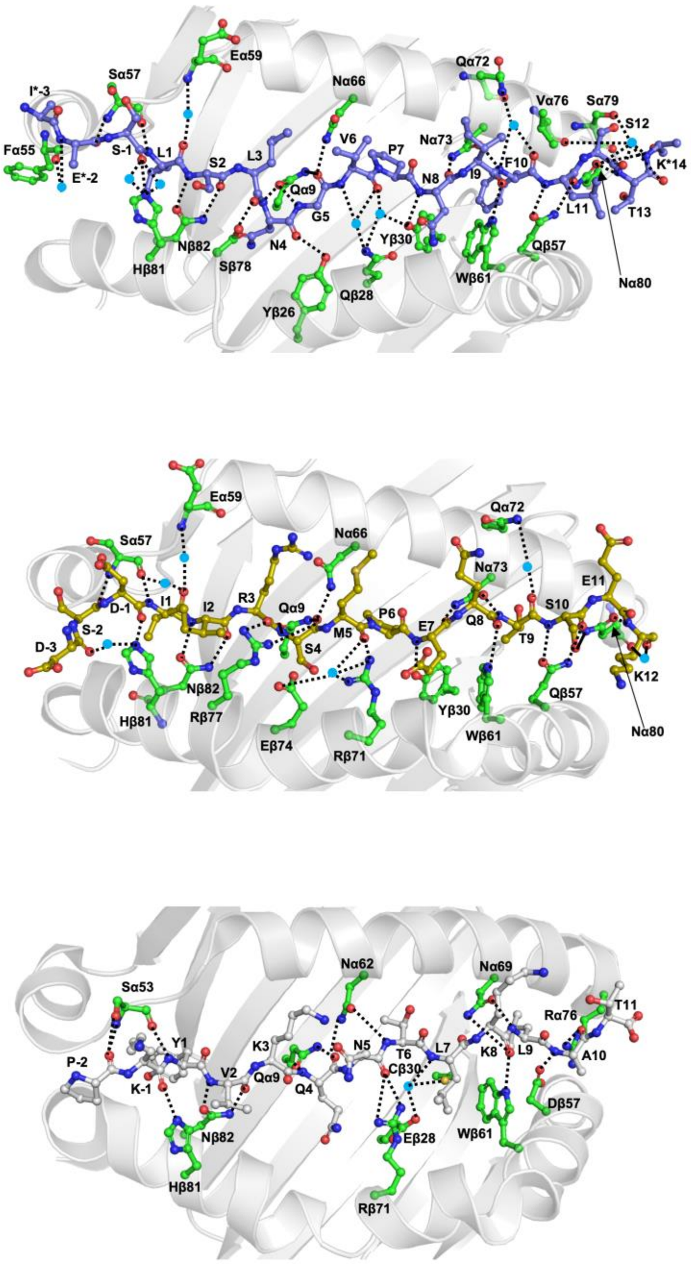

S7 Fig. 3D representation of H-bonds (dotted lines, cut-off of 4 Å) between class II molecules (ribbons with side chains of key interacting residues in sticks), peptide in sticks, and waters as blue circles. Top panel, BL2*02 (6T3Y) with peptide in blue; middle panel, BL2*19 (6KVM) with peptide in yellow; bottom panel, HLA-DR1*01 (1DLH) with peptide in grey. Based on LigPlot analyses in S5 Fig. The underlying data for this figure can be found in PDB files 1DLH, 4X5W, 6KVM and 6T3Y.
