# Supplementary material for: The dominantly expressed class II molecule from a resistant MHC haplotype presents only a few Marek’s disease virus peptides by using an unprecedented binding motif
Source: PLoS Biol. 2021 Apr 26;19(4):e3001057. doi: 10.1371/journal.pbio.3001057 (PMC8101999; doi:10.1371/journal.pbio.3001057)
Supplement: S8 Fig — Schematic of H-bonds interacting with peptide main chain atoms (cut-off of 4 Å) comparing HLA-DR1*01 (solid lines), BL2*19 (dashed lines), and BL2*02 (dotted lines), including H-bonds through waters (solid blue circles), based on structures 1DLH for DR1*01, 6KVM for BL*19, and 6T3Y for BL*2 analyzed by LigPlot (S6 Fig). The underlying data for this figure can be found in PDB files 1DLH, 4X5W, 6KVM, and 6T3Y. (PDF) [file pbio.3001057.s008.pdf]

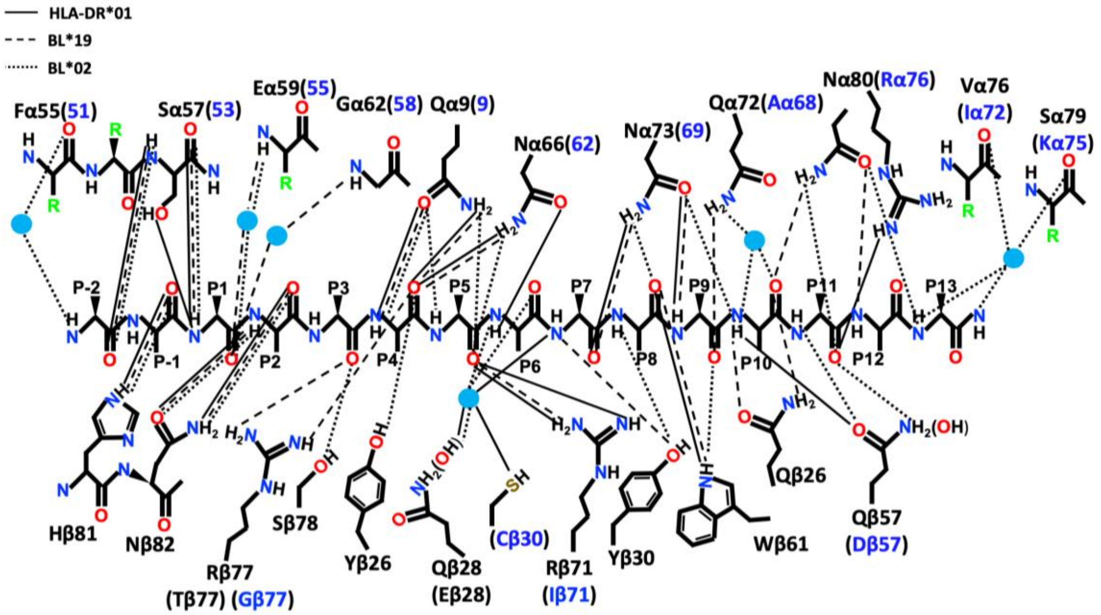

S8 Fig. Schematic of H-bonds interacting with peptide main chain atoms (cut-off of 4 Å) comparing HLA-DR1*01 (solid lines), BL2*19 (dashed lines) and BL2*02 (dotted lines), including H-bonds through waters (solid blue circles), based on structures 1DLH for DR1*01, 6KVM for BL*19 and 6T3Y for BL*2 analyzed by LigPlot (S5 Fig). The underlying data for this figure can be found in PDB files 1DLH, 4X5W, 6KVM and 6T3Y.
