# Supplementary material for: The dominantly expressed class II molecule from a resistant MHC haplotype presents only a few Marek’s disease virus peptides by using an unprecedented binding motif
Source: PLoS Biol. 2021 Apr 26;19(4):e3001057. doi: 10.1371/journal.pbio.3001057 (PMC8101999; doi:10.1371/journal.pbio.3001057)
Supplement: S9 Fig — Dot plots (GFP from MDV on x-axis, PE from antibody staining on the y-axis) for (A) chicken class II molecules using the monoclonal antibody 2G11 and (B) isotype control antibody. (C) Histogram of antibody staining (mean fluorescent intensity on x-axis, number of events on the y-axis); red, 2G11 staining; blue, isotype control. CECs, chicken embryo cells; GFP, green fluorescent protein; MDV, Marek’s disease virus; PE, phycoerythrin; TEV, tobacco etch virus. (PDF) [file pbio.3001057.s009.pdf]

A.

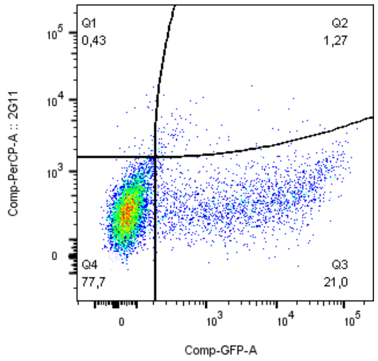

B.

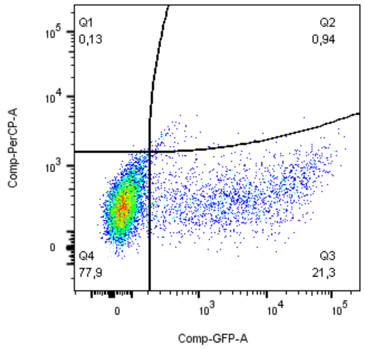

C.

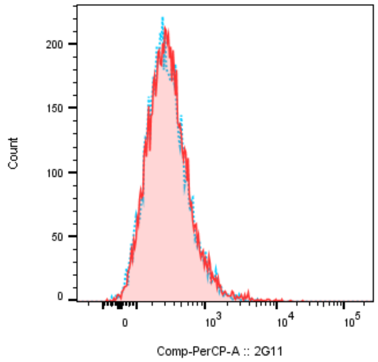

S9 Fig. Flow cytometric analysis of chicken embryo cells (CECs) used for infection of bursal B cells shows virtually no expression of chicken class II molecules. Dot plots (GFP from MDV on x-axis, PE from antibody staining on the y-axis) for A. chicken class II molecules using the monoclonal antibody 2G11) and B. isotype control antibody. C. Histogram of antibody staining (mean fluorescent intensity on x-axis, number of events on the y-axis); red, 2G11 staining; blue, isotype control.
