# Supplementary material for: The dominantly expressed class II molecule from a resistant MHC haplotype presents only a few Marek’s disease virus peptides by using an unprecedented binding motif
Source: PLoS Biol. 2021 Apr 26;19(4):e3001057. doi: 10.1371/journal.pbio.3001057 (PMC8101999; doi:10.1371/journal.pbio.3001057)
Supplement: S10 Fig — Sequences in single letter amino acid code, all black except: bold blue, TEV protease cleavage site; bold oranges, c-fos tag; bold gold, c-jun tag; bold red, V5-tag; normal red, Avi-tag (biotinylation site). Highlights: green, GP67 signal sequence; deep pink, peptides; cyan, linkers; grey, MHC extracellular domains; yellow, 6xHis-tag. MHC, major histocompatibility complex; TEV, tobacco etch virus. (PDF) [file pbio.3001057.s010.pdf]

>BLA
MLLVNQSHQGFNKEHTSKMVSAIVLYVLLAAAAHSAFAADRRHVLLQAEFYQRSEGPDKAWA
QFGFHFDADELFHVELDAAQTVWRLPEFGRFASFEAQGALQNMAVGKQNLEVMISNSNRSQ
QDFVTPELALFPAEAVSLEEPNVLICYADKFWPPVATMEWRRNGAVVSEGVYDSVYYGRPDL
LFRKFSYLPFVPQRGDVYSCAVRHWGAEGPVQRMWEPEVPEPPSEGGGSRSGGGSTDTLQ
AETDQLEDEKSALQTEIANLLKEKEKLEFILAAYGGGSGGGSGTGLNDIFEAQKIEWHE--

>gE-peptide_BLB2
MLLVNQSHQGFNKEHTSKMVSAIVLYVLLAAAAHSAFAADRPQIESLSLNGVPNIFLSTKAGGG
GSGGGGSGGGGSSAFFFCGAIFECHYLNGTERVRYLQRYIYNRQQLVHFDSDVGKFVADTPL
GEPQAEYWNSNAELLENIMNIADGSCRHNYGILESFTVQRSVEPKVRVSALQSGSLPETDRLA
CYVTGFYPPEIEVKWFLNGREETERVVSTDVMQNGDWTYQVLVVLETVPRRGDSYVCRVEH
ASLRQPISQAWEPPADAGRSKGENLYFQGGSIARLEEKVKTLKAQNSELASTANMLREQVA
QLKQKVMNGGGSGGGSSRGPFEGKPIPNPLLGLDSTRTGHHHHHH-

>gH-peptide_BLB2
MLLVNQSHQGFNKEHTSKMVSAIVLYVLLAAAAHSAFAADRGVLFYMPTSHVQQMTFGGGGS
GGGGSGGGGSSAFFFCGAIFECHYLNGTERVRYLQRYIYNRQQLVHFDSDVGKFVADTPLGE
PQAEYWNSNAELLENIMNIADGSCRHNYGILESFTVQRSVEPKVRVSALQSGSLPETDRLACY
VTGFYPPEIEVKWFLNGREETERVVSTDVMQNGDWTYQVLVVLETVPRRGDSYVCRVEHASL
RQPISQAWEPPADAGRSKGENLYFQGGSIARLEEKVKTLKAQNSELASTANMLREQVAQLK
QKVMNGGGSGGGSSRGPFEGKPIPNPLLGLDSTRTGHHHHHH-

>UL43-peptide_BLB2
MLLVNQSHQGFNKEHTSKMVSAIVLYVLLAAAAHSAFAADRSSEVLTSIGKPAQFIFAGGGGS
GGGGSGGGGSSAFFFCGAIFECHYLNGTERVRYLQRYIYNRQQLVHFDSDVGKFVADTPLGE
PQAEYWNSNAELLENIMNIADGSCRHNYGILESFTVQRSVEPKVRVSALQSGSLPETDRLACY
VTGFYPPEIEVKWFLNGREETERVVSTDVMQNGDWTYQVLVVLETVQRRGDSYVCRVEHAS
LRQPISQAWEPPADAGRSKGENLYFQGGSIARLEEKVKTLKAQNSELASTANMLREQVAQL
KQKVMNGGGSGGGSSRGPFEGKPIPNPLLGLDSTRTGHHHHHH-

>gI-peptide_BLB2
MLLVNQSHQGFNKEHTSKMVSAIVLYVLLAAAAHSAFAADRTPSDVIEKELMEKLKKKGGGGS
GGGGSGGGGSSAFFFCGAIFECHYLNGTERVRYLQRYIYNRQQLVHFDSDVGKFVADTPLGE
PQAEYWNSNAELLENIMNIADGSCRHNYGILESFTVQRSVEPKVRVSALQSGSLPETDRLACY
VTGFYPPEIEVKWFLNGREETERVVSTDVMQNGDWTYQVLVVLETVPRRGDSYVCRVEHASL
RQPISQAWEPPADAGRSKGENLYFQGGSIARLEEKVKTLKAQNSELASTANMLREQVAQLK
QKVMNGGGSGGGSSRGPFEGKPIPNPLLGLDSTRTGHHHHHH-

S10 Fig. Protein sequences encoded by constructs for soluble class II chains in Baculoviruses. Sequences in single letter amino acid code, all black except: bold blue, tobacco etch virus (TEV) protease cleavage site; bold oranges, c-fos tag; bold gold, c-jun tag; bold red, V5-tag; normal red, Avi-tag (biotinylation site). Highlights: green, GP67 signal sequence; deep pink, peptides; cyan, linkers; grey, MHC extracellular domains; yellow, 6xHis-tag.
