# Supplementary material for: The dominantly expressed class II molecule from a resistant MHC haplotype presents only a few Marek’s disease virus peptides by using an unprecedented binding motif
Source: PLoS Biol. 2021 Apr 26;19(4):e3001057. doi: 10.1371/journal.pbio.3001057 (PMC8101999; doi:10.1371/journal.pbio.3001057)
Supplement: S1 Table — The underlying data for this figure can be found in Fig 2 and S1–S6 Data. (PDF) [file pbio.3001057.s011.pdf]

S1 Table. Peptides from class II molecules of bursal B cells infected in vitro. The underlying data for this figure can be found in Table 1 and S1-S6 Data.

gene	protein	
MDV022/UL10	gM	Q9E6Q6: envelope glycoprotein (although no glycosylation sites indicated in UniProt) with 8 TM regions, 424 aa, forms disulfide-linked dimer with gN, important for virion assembly and egress, and incorporation of gH-gL into viron membrane
MDV025/UL13		Q9E6Q4: serine/threonine kinase, 513 aa, involved in egress of viral particles from nucleus, modulation of actin cytoskeleton and regulation of viral and host gene expression
MDV030/UL18	TRX2	Q9E6P9: triplex capsid protein 2 (TRX2), 319 aa, two TRX2 with one TRX1 make trimers which link hexons and pentons in capsid
MDV031/UL19	MCP	Q9E6P8: major capsid protein (MCP), 1393 aa, self-assembles into 150 hexons and 12 pentons to make icosahedral capsid, surrounded by tegument
MDV033/UL21		Q9E6P7: tegument protein UL21 homolog, 546 aa, may facilitate viral transport in nerves
MDV034/UL22	gH	Q9E6P6: glycoprotein with 1 TM on viral membrane, 813 aa, as heterodimer with gL and together with gB mediates membrane fusion
MDV040/UL27	gB	Q77MS3: envelope glycoprotein with 1 TM, 865 aa, cleaved into two disulfide-linked parts by furin, forms spike for heparan sulfate binding essential for initial virion binding and then in fusion along with gH/gL
MDV046/UL32		Q9E6N7: packaging protein UL32 homolog, 41 aa, involved in localisation of newly-synthesised capsids to nuclear replication compartments
MDV056/UL43		Q9E6M9: membrane protein UL43 homolog with 11 TM segments, 420 aa, found in viral tegument but no function in UniProt, not well conserved
MDV057/UL44	gC	P22651: envelope glycoprotein with 1 TM with Ig-like domain as GP57-65 or Glycoprotein A but predominantly secreted, 501 aa, involved in early stage immunosuppression?
MDV062/UL49	VP22	Q9E6M7: highly phosphorylated tegument protein in complex with VP16 and UL41/VHS, 249 aa, interacts with gE and gM, highly abundant at 2000 copies per virion
MDV066/UL52		Q9E6M4: nuclear DNA primase with one Zn finger, 1074 aa, essential component of helicase/primase complex
MDV070/UL55		Q77MR1: tegument protein UL55 homolog, 166 aa, no function described in UniProt
MDV078	vIL-8	Q77MQ6: viral IL-8 homolog, chemokine, 134 aa, attracts B cells (and T cells?)
MDV082		Q9DH37: uncharacterised gene, 110 aa, no obvious features in UniProt
MDV095/US7	gI	Q9E6L5: envelope TM glycoprotein, 35 aa, with gE required for cell-to-cell spread
MDV096/US8	gE	Q77MP7: envelope glycoprotein with 1 TM and with phosphoryation, 497 aa, as heterodimer with gI required for cell-to-cell spread by localisation to cell junctions, also basolateral spread in polarised epithelia and anterograde spread in nerves
