# Supplementary material for: The dominantly expressed class II molecule from a resistant MHC haplotype presents only a few Marek’s disease virus peptides by using an unprecedented binding motif
Source: PLoS Biol. 2021 Apr 26;19(4):e3001057. doi: 10.1371/journal.pbio.3001057 (PMC8101999; doi:10.1371/journal.pbio.3001057)
Supplement: S2 Table — The underlying data for this figure can be found in PDB file 6T3Y. (PDF) [file pbio.3001057.s012.pdf]

S2 Table. Crystallographic statistics. The underlying data for this figure can be found in PDB file 6T3Y.

**BL2*02 (6T3Y)**	
**Data collection and statistics**	
**Beamline**	I04-1
**Wavelength (Å)**	0.978
**resolution range**	26.55-1.70 (1.76-1.70)
**space group**	C 1 2 1
**unit cell**	154.15 60.00 51.71 90 108.75 90
**total reflections**	294236 (29547)
**unique reflections**	49286 (4917)
**multiplicity**	6.0 (6.0)
**completeness (%)**	99.75 (99.59)
**mean I/sigma (I)**	18.39 (2.26)
**Wilson B-factor**	21.85
**R-merge**	0.052 (0.71)
**R-meas**	0.057 (0.78)
**R-pim**	0.023 (0.31)
**CC1/2**	1.0 (0.88)
**CC***	1.0 (0.97)
**Refinement and validation**	
**total reflections**	49209 (4899)
**reflections used for R-free**	4968 (470)
**R-work**	0.17 (0.28)
**R-free**	0.20 (0.32)
**CC(work)**	0.97 (0.92)
**CC(free)**	0.96 (0.88)
**number of non-hydrogen atoms**	3366
**macromolecules**	3027
**ligands**	22
**solvent**	317
**protein residues**	379
**RMS(bonds)**	0.01
**RMS(angles)**	1.13
**Ramachandran favored (%)**	98.37
**Ramachandran allowed (%)**	1.63
**Ramachandran outliers (%)**	0.00
**rotamer outliers (%)**	0.00
**Clashscore**	5.60
**average B-factor**	35.61
**macromolecules**	34.75
**ligands**	66.43
**solvent**	41.68
**number of TLS groups**	10
