# Supplementary material for: The dominantly expressed class II molecule from a resistant MHC haplotype presents only a few Marek’s disease virus peptides by using an unprecedented binding motif
Source: PLoS Biol. 2021 Apr 26;19(4):e3001057. doi: 10.1371/journal.pbio.3001057 (PMC8101999; doi:10.1371/journal.pbio.3001057)
Supplement: S3 Table — The underlying data for this figure can be found in PDB files 1DLH, 4X5W, 6KVM, and 6T3Y. (PDF) [file pbio.3001057.s013.pdf]

S3 Table. Root mean square deviation (RSMD) of BL2*02 (6T3Y) against chains and domains from three other class II structures. The underlying data for this figure can be found in PDB files 1DLH, 4X5W, 6KVM and 6T3Y.

Protein	PDB ID	Overall Structure	α-chain	α1-domain	α2-domain	β-chain	β1-domain	β2-domain
HLA-DR1*01	1DLH	0.878	0.737	0.341	0.591	0.787	0.782	0.407
BL2*19	6KVM	0.684	0.565	0.228	0.327	0.588	0.554	0.304
HLA-DR1*01	4X5W	0.930	0.829	0.378	0.397	0.810	0.731	0.352
