# Supplementary material for: The dominantly expressed class II molecule from a resistant MHC haplotype presents only a few Marek’s disease virus peptides by using an unprecedented binding motif
Source: PLoS Biol. 2021 Apr 26;19(4):e3001057. doi: 10.1371/journal.pbio.3001057 (PMC8101999; doi:10.1371/journal.pbio.3001057)
Supplement: S4 Table — (PDF) [file pbio.3001057.s014.pdf]

S4 Table. Amino acid residues of BL*02 (6T3Y), BL*19 (6KVM), and HLA-DR1 (1DLH and 4X5W) that form H-bonds to the main chain of the peptide as determined by LigPlot+, with a maximum hydrogen-acceptor distance of 2.70 Å, a maximum donor-acceptor distance of 3.35 Å, and minimum and maximum contact distances for hydrophobic residue to any contact of 2.90 Å and 3.90 Å,respectively. Bridging water molecules are designated by "=HOH". The underlying data for this figure can be found in PDB files 1DLH, 4X5W, 6KVM and 6T3Y.

**BL*02 (6T3Y) Peptide**	BL*02 (6T3Y)	**BL*19 (6KVM) Peptide**	BL*19 (6KVM)	**HLA-DR1 (1DLH) Peptide**	HLA-DR1 (1DLH)	**HLA-DR1 (4X5W) Peptide**	HLA-DR1 (4X5W)
		**P-5 (P)**	---				
		**P-4 (G)**	---				
**P-3 (I)**	---	**P-3 (D)**	Hβ81=HOH			**P-3 (V)**	Sα53=HOH
**P-2 (E)**	Sα57, Fα55=HOH	**P-2 (S)**	Sα57	**P-2 (P)**	Sα53	**P-2 (S)**	Sα53, Fα51=HOH
**P-1 (S)**	Hβ81	**P-1 (D)**	Hβ81	**P-1 (K)**	Hβ81	**P-1 (K)**	Hβ81
**P1 (L)**	Sα57, Eα59=HOH	**P1 (I)**	Sα57, Eα59=HOH	**P1 (Y)**	Sα53	**P1 (W)**	Sα53, Eα55=HOH
**P2 (S)**	Nβ82	**P2 (I)**	Nβ82	**P2 (V)**	Nβ82	**P2 (R)**	Nβ82
**P3 (L)**	---	**P3 (R)**	Rβ77, Rβ77=HOH	**P3 (K)**	---	**P3 (M)**	---
**P4 (N)**	Qα9, Yβ26	**P4 (S)**	Qα9, Nα66	**P4 (Q)**	Qα9, Nα62	**P4 (A)**	Qα9, Nα62
**P5 (G)**	Qα9, Nα66	**P5 (M)**	Rβ71, Rβ71=HOH, Eβ74=HOH	**P5 (N)**	Rβ71	**P5 (T)**	Rβ71, Nα62=HOH
**P6 (V)**	Qβ28=HOH	**P6 (P)**	---	**P6 (T)**	---	**P6 (P)**	---
**P7 (P)**	---	**P7 (E)**	Yβ30, Nα73	**P7 (L)**	Nα69	**P7 (L)**	Nα69, Eβ28=HOH
**P8 (N)**	Yβ30, Nα73	**P8 (Q)**	Wβ61	**P8 (K)**	Wβ61	**P8 (L)**	Wβ61, Qβ64=HOH
**P9 (I)**	Wβ61	**P9 (T)**	Nα73	**P9 (L)**	Nα69	**P9 (M)**	Nα69, Yβ60=HOH
**P10 (F)**	Nα73, Nα73=HOH, Qα72=HOH	**P10 (S)**	Qβ57, Nα80	**P10 (A)**	Dβ57	**P10 (Q)**	Dβ57=HOH, Rα76=HOH
**P11 (L)**	Qβ57, Nα80	**P11 (E)**	---	**P11 (T)**	---	**P11 (A)**	---
**P12 (S)**	---	**P12 (K)**	Nα80			**P12 (L)**	---
**P13 (T)**	Nα80	**---**					
**P14 (K)**	Sα79=HOH, Nα80=HOH	**---**					
