# Supplementary material for: The dominantly expressed class II molecule from a resistant MHC haplotype presents only a few Marek’s disease virus peptides by using an unprecedented binding motif
Source: PLoS Biol. 2021 Apr 26;19(4):e3001057. doi: 10.1371/journal.pbio.3001057 (PMC8101999; doi:10.1371/journal.pbio.3001057)
Supplement: Attachment — Submitted filename: Response to Reviewers - Halabi et al PLoS Biol 12Mar21.docx [file pbio.3001057.s021.docx]

Response to Reviewers for Halabi et al for PLoS Biology, 12Mar21

Previously entitled "Differential host response to a herpesvirus: Marek’s disease virus peptides on chicken MHC class II molecules are derived from only a few genes and illustrate a new mode of peptide binding"

but now with a revised title “The dominantly-expressed class II molecule from a resistant MHC haplotype presents only a few Marek’s disease virus peptides by using an unprecedented binding motif”.

Reviewer #1: Immunopetidomics.

*Reviewer #1: Here Halabi et al present a study characterising peptide presentation by the chicken MHC class II of the B2 haplotype in a Marek's disease virus (MDV) infection model, incorporating both immunopeptidomics and structural biology. Whilst mammalian MHC class II have been broadly studied, data on chicken MHC II are comparatively sparse. Only a handful of studies have utilised mass spectrometry to characterise peptide presentation by chicken MHCII, revealing relatively few ligands. Furthermore, only one structure of a chicken MHCII (BL2*19) has been published prior to this study. Thus, Halabi et al. greatly advance knowledge of peptide presentation by chicken MHCII, identifying over 8000 naturally presented peptides through LC-MS/MS analysis of peptides eluted from immunoaffinity purified MHCII, and pairing these data with structural characterisation of BL2*02 to define the MHCII binding motif. Moreover, combining these techniques, they reveal novel MDV-derived ligands and a novel mode of MHCII binding. These data will be of great interest for vaccine development in the poultry industry, as well as to those in broader immunological research, from both an evolutionary and mechanistic perspective of antigen presentation.*

Response: We thank the reviewer for the positive comments, which we believe accurately describe the contributions that this paper will make.

*Major comments:*

*1. Gibbs clustering is used to dissect the binding motifs of the co-expressed BL1 and BL2 and lines 157 and 158 state "the ratio of peptides from these ex vivo bursal B cells was around 40% nonamer and 60% decamer in all samples (Datasets S1-S6)."*

*However, whilst table 1 notes which MDV peptides were assigned to either cluster 1 or cluster 2, this is not shown in the full Datasets S1-S6, making it hard to determine the ratio as stated.*

*Nor are the numbers of peptides on which each motif is generated shown in Figure 1.*

*To enable evaluation by the reader, further details on the parameters used for Gibbs clustering (including pre-processing such as whether overlapping peptide species were collapsed into single epitopes prior to clustering, if datasets were analysed by gibbs clustering individually or collectively) should be incorporated into the methods.*

*Assignment to cluster 1 or 2 could be added to the Datasets file, and/or the number of peptides on which the motifs were generated added to figure 1.*

Response: We have added additional description of the analysis by Gibbs clustering to the methods section, as follows: “Possible motifs for peptides of length 10 amino acids or greater were grouped using GibbsCluster-2.0 [70], specifying parameters for class II ligands, number of groups, length of motif and first residue as a hydrophobic amino acid; the process collapses all species with post-translational modifications like oxidized methionines to one entry, but keeps any overlapping or nested sequences as separate entries.”

We have added columns to each of the six Datasets describing the motifs found by Gibbs clustering for each of the 18,434 peptides, as well as tables and Venn diagrams with both peptide numbers and percentages. We have also rechecked the motifs of each of the peptide species listed in Table 1 (having found a few minor changes compared to the previous analyses), and underlined the motifs for the four peptides described in the legend to Fig. 1.

While these results are consistent with the previous statement that "the ratio of peptides from these ex vivo bursal B cells was around 40% nonamer and 60% decamer in all samples”, the sentence has been rewritten to more clearly state the findings: “However, the peptide species from these *ex vivo* bursal B cells that contain the BL1 nonamer motif ranged from 37-48% of the total containing BL1 and BL2 motifs in different samples (Datasets S1-S6).”

To be clear, these reanalyses led to a few changes in the motifs for a few MDV peptides, leading to changes in the revised version of Table 1, none of which impact the conclusions of the paper (except that a greater proportion of MDV peptides were assigned to the BL2*02 motif). We are not able to explain why the Gibbs clustering function might change motif assignment from “both” to “1” or “2”, or from “1” or “both” to “neither”.

*2. For infection, a co-culture system is used in which there are 3 infected CECs to every 100 B2/B2 bursal cells.*

*Has MHCII expression by the CECs been considered/controlled for?*

*Could this be contributing to peptides isolated, thus impacting the motif characterisation?*

*Is the MHC haplotype of the CECs known?*

Response: We had indeed checked the potential contribution by CECs, and found that they do not express class II molecules so are not expected to contribute to the peptides found. As mentioned in the Materials and Methods, the CECs were derived from embryos of the VALO line, which we have since determined segregate B15, B19 and B21 MHC haplotypes. However, given that the CECs do not express class II molecules, this information is not likely to be germane. We have added a new figure (Fig. S9) with flow cytometric analysis of the CECs to the Supplementary Information.

*Minor comments*

*3. In the legend for figure 1, it would be useful to indicate the region of each peptide that is considered to be the decamer or nonamer motif*

Response: Done.

*4. For clarity, would it be appropriate to label gH in Figure 2, for those not familiar with the virus like myself? Or include UL22 in the main text?*

Response: Many thanks for spotting this inadvertent inconsistency. The label UL22 has been changed to gH on the figure. Since there are several nomenclatures used for herpesvirus genes and proteins, UL22 and gH are mentioned together both in the text and in Table 1.

*5. In table 1, are any of the detected peptides unique to a given strain? It is not currently commented upon whether all peptides are within the proteomes of both viral strains used.*

Response: As can be seen from Table 1, the vaccine strain CVI988 had many peptides not found for RB1B. This point is now noted by an additional sentences: “Many of these peptide species and/or epitopes are shared between the live attenuated virus strain CVI988 and the very virulent MDV strain RB1B, with many other peptides unique to CVI988 but none unique to RB1B. The infection rate was much higher for CVI988 than for RB1B in these *in vitro* cultures, which may have resulted in more class II molecules being loaded with MDV peptides.”

*6. In table 1 and the supplementary datasets, are lower case "m"s oxidised methionine? Could this be defined?*

Response: Lower case “m”s are indeed oxidised methionines. This usage is now defined in each of the datasets, and changed to “M”s in Table 1.

*7. Line 261 - structure is missing a c*
*8. Line 323 - I only counted 9/23, rather than 10/23?*
*9. Line 432 - "Analysis of peptides by LC- and below MS/MS." Should "and below" be removed?*

Response: Many thanks for spotting these inadvertent errors, which are now corrected.

*10. Line 442 - the mass range is stated as 400-650m/z, precursors 2+ and 3+. This seems a bit narrow. Should 650 read 1650?*

Response: Again our thanks for spotting this inadvertent error, which arose from concurrent work with class I peptides. The sentence is now corrected to read “The mass range was limited to 300–1500 m/z with precursors of charge states greater than or equal to 2 eligible for fragmentation.”

Reviewer #2: Structural immunology.

*Reviewer #2: Unlike their human counterparts, chicken MHC class II genes have strong associations with infectious disease and are thus of critical importance for the poultry industry. A major poultry pathogen is an oncogenic herpes virus that causes Marek's disease and has a genome comprising >100 genes. The B2 MHC haplotype correlateds with susceptibility to Marek's. Using new technology, Halabi et al have have comprehensively characterized the peptides that are bound by the MHC class II isoforms encoded by the B2 haplotype. The vast majority of these peptides derive from the products of only four genes, and most are presented by BL2, the dominant MHC class II of the chicken. Whereas most MHC class II isoforms engage a nonamer sequence within an antigenic peptide, BL2 engages a decamer. Crystallographic analysis shows that BL2 engages a decamer. This is achieved by a small amino acid causing a crinkle in the peptide.

This manuscript has the potential to make a valuable contribution to the immunology of the domestic chicken and its many fascinating differences form the human counterpart.

The main weakness is the manuscript, which is too long, poorly structured and as a consequence impedes readers' comprehension.*

Response: We thank the reviewer for the clear description of the paper and of the potential contribution (although we should mention that it is stated several times in the manuscript that the B2 haplotype confers resistance, not susceptibility, to Marek’s disease).

However, we are somewhat at a loss to respond to the sweeping characterisation of the manuscript being too long and poorly structured, given that there is no detailed description of what the reviewer considers to be at fault.

We agree that the paper involves several areas that are not found frequently in PLoS Biology papers and could present challenges to some readers, including a poultry disease, the MHC, immunopeptidomics and structural biology. Therefore, every sentence was chosen with this thought in mind, with an extensive introduction and reference list to allow a diverse readership to appreciate each aspect of the paper. We shortened small parts of the introduction, but otherwise were unable to find excess verbiage or unimportant points in this manuscript, and indeed were required to add a few sentences clarifying points raised by the first reviewer.

Given the challenge of conveying the messages clearly, the manuscript was structured in a simple and logical way:

Introduction including the importance of viral disease, the strong genetic associations of the chicken MHC with infectious disease, MDV and MHC resistance, structure of class II molecules, statement of the question to be answered by the paper, and a summary of what was done (seven paragraphs),

Results 1. the general results of the immunopeptidomics which describe for the first time peptide motifs outside of mammals including the unprecedented 10mer core sequence (three paragraphs and part of Fig. 1),

Results 2. the MDV peptides found by immunopeptidomics including the completely unexpected result of only a few MDV genes giving rise to the majority of the peptides (four paragraphs, Table 1 and part of Fig. 1),

Results 3. the overall results of the structural biology showing the conserved features of the chicken class II molecule (two paragraphs and Fig. 2),

Results 4. the detailed description of the interaction of the peptide with the MHC molecule which explains the structural mechanism leading to the 10mer core sequence (three paragraphs and Fig. 3), and

Discussion to summarise the surprising results, compare to the wider literature, and describe the potential of the findings (seven paragraphs).

We were not able to identify a better way to organise the manuscript.

Given our difficulties in identifying serious problems with length and organisation, we sent this first manuscript to five researchers spanning generations and areas of expertise (including evolutionary biology, cell biology, developmental biology, cellular immunology and structural biology). All of them responded with comments that the manuscript was relatively easy to understand, but each suggested a few small details to improve the manuscript, which we incorporated.
